# Model of B_9_N_9_ Response under External Electric Field: Geometry, Electronic Properties, Reaction Activity

**DOI:** 10.3390/molecules27051714

**Published:** 2022-03-06

**Authors:** Xupu Wu, Dasen Ren

**Affiliations:** 1 School of Mechatronics Engineering, Guizhou Minzu University, Guiyang 550025, China; aswxp23@163.com; 2 School of Physics and Electronic Sciences, Guizhou Education University, Guiyang 550018, China

**Keywords:** B_9_N_9_, external electric field, polarization, electrophilic reaction, nucleophilic reaction

## Abstract

In this paper, we performed the ωB97XD/def2-TZVP method with a density functional theory study on the boron–nitrogen (BN) analogues of cyclo[18]carbon. The geometric structure, polarization properties, and excitation effect were calculated in the presence of an external electric field (EEF). Furthermore, the dual descriptor and Fukui function matrices were employed to predict the tendency towards the electrophilic or nucleophilic reactions of B_9_N_9_ under varying EEF strengths. The results show that the application of an EEF will cause the cyclic structure of B_9_N_9_ to be considerably distorted towards an elliptical geometry, the polarization to increase, and the reactivity of B_9_N_9_ to enhance with the increase in the EEF strength. This is of great significance for further experimental exploration into the catalytic properties of BN fullerenes.

## 1. Introduction

Since 1980, boron nitride (BN) nanotubes and BN clusters analogous to carbon-based fullerenes have been synthesized [1,2,3]. These BN fullerene materials are found to have excellent thermal conductivity [4], stable chemical properties [5], and excellent catalytic ability [6], so they are widely used in biomedical materials [7], catalysis, and other related fields. In 2019, the first cyclo[18]carbon material was synthesized and characterized under a high-resolution atomic force microscope, demonstrating a unique geometric configuration and electronic structure [8]. Theoretical studies showed that the cyclo[18]carbon structure has excellent electronic [9,10,11] and chemical properties [12,13], and could have potential applications in the field of electronic devices. Boron, in main group III, has one valence electron less than carbon, in main group IV, whereas nitrogen, in main group V, has one valence electron more than carbon. Therefore, B_9_N_9_, with alternating B and N atoms, is isoelectronic with cyclo[18]carbon. The optimization calculation shows that the geometric structure of B_9_N_9_ is similar to the structure of cyclo[18]carbon, and they display many properties similar to each other. The similarity of B_9_N_9_ to cyclo[18]carbon in structure and its desirable properties and promising applications have attracted the widespread attention of researchers [14,15,16]. Despite the chemical inertness characteristic of BN materials under conventional circumstances [5], these materials possess extraordinary catalytic properties [6,12,17]. J. T. Grant [6] suggested that the active site of BN nanomaterials as catalysts switches between B and N, depending on the reaction conditions. Zhen Liu et al. showed that the dissociation of B-N bonds leads to the formation of active radicals in BN materials [12]; the acceleration of the dissociation of B–N bonds would help to improve the catalytic performance of BN materials.

External electric fields (EEF) usually change the internal and external electronic environments of molecules [18,19] and disrupt their geometric structure [20,21,22,23]. It is expected that EEF will have a strong influence on B_9_N_9_ geometry, electrons, and reactivity properties. In this paper, we will investigate the changes in the geometric and the electronic structure of B_9_N_9_ under different EEF strengths, and finally make predictions regarding the location of the B_9_N_9_ reactive sites and the tendency for electrophilic or nucleophilic substitution. We also discuss the effect of adding alkali metal and alkaline earth metal ions near the B_9_N_9_ molecule on the molecular geometry and absorption spectra.

## 2. Methods

All electronic structure calculations of B_9_N_9_ were performed within density functional theory (DFT) using the ωB97XD exchange–correlation function [24,25]. The results calculated in this work using ωB97XD/def2-TZVP [26] agree well with the structures calculated by Fabio [14] (see Table 1).

**Figure 1:**
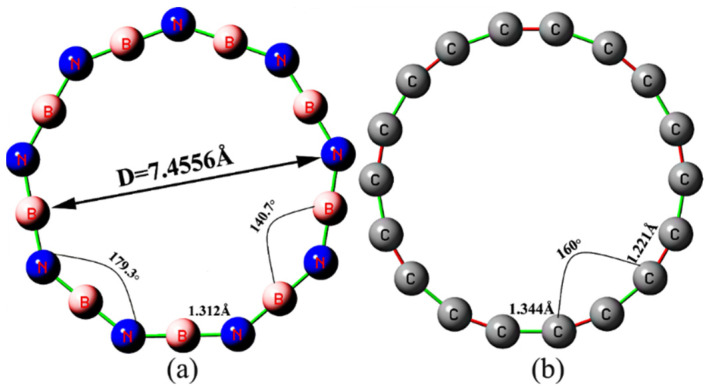
Structure of the optimized B_9_N_9_ (**a**) and cyclo[18]carbon (**b**) molecule based on ωB97XD/def2-TZVP calculations.

As shown in Table 1 and Figure 1, B_9_N_9_ possesses a cyclic structure consisting of nine B atoms alternating with nine N atoms, with identical bond lengths between atoms and alternating bond angles between B-N-B and N-B-N, thus forming a cyclic structure consisting of nine BN units in the plane. From the viewpoint of molecular geometry, B_9_N_9_ and cyclo[18]carbon have certain similarities. However, B_9_N_9_ consists of two elements, leading to alternating angles of 140.7° and 179.3° for B-N-B and N-B-N, respectively, with a B-N bond length of 2; whereas cyclo[18]carbon is a ring with an angle of 160° consisting of alternating C-C(1.344 Å) long bonds and C-C(1.221 Å) short bonds. These effects lead to some differences in the variation of their performance under the action of the external electric field.

In this work, all geometric structures were obtained in the absence of symmetry constraints, without imaginary frequencies, corresponding to a small part of the potential energy surface. The direction of the applied EEF is in the y-direction, for reasons that will be explained in Section 3.1. All calculations were performed using Gaussian16 [27]. Based on the Gaussian16 output file, the relevant parameters extracted by the program Multiwfn3.8 [28] were used, and then plots such as electrostatic potential distribution and hyperpolarization visualization were drawn using VMD1.9.3 [29]. The notation convention for EEF in Gaussian programs is opposite to the general physical definition; in this paper, we followed the same EEF convention as the general physical definition, in which 1 a.u. of EEF corresponds to 51.4 V/A, and 1 V/A is approximately equal to 0.02 a.u.

## 3. Results and Discussion

### 3.1. Effect of an EEF on the Geometric Structure of B_9_N_9_

**Figure 2:**
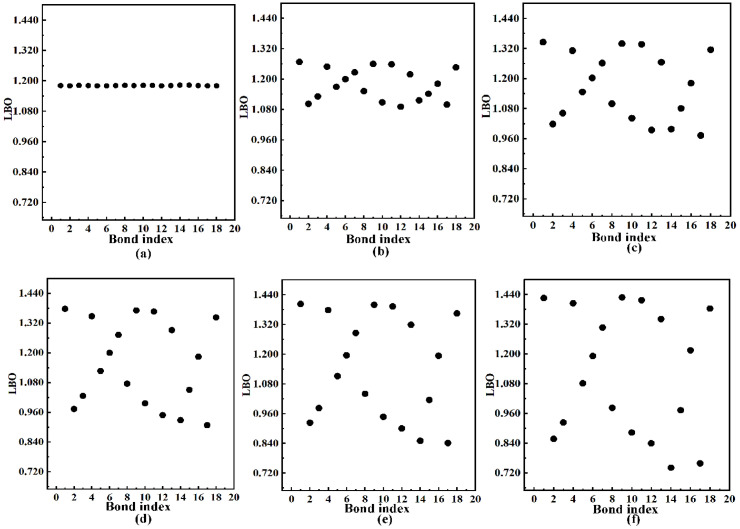
The figure shows the variation in LBO of B_9_N_9_ under different EEF intensities. The EEFs corresponding to (**a**–**f**) are 0 a.u., 0.01 a.u., 0.02 a.u., 0.025 a.u., 0.03 a.u., and 0.0365 a.u., respectively.

Figure 2 shows a 2D scatter plot of the change in Laplacian bond order (LBO) [30] of B_9_N_9_ in the presence of EEF. The EEF can effectively change the geometry of B_9_N_9_; the stronger the electric field, the greater the deformation. In the absence of an electric field, the B_9_N_9_ molecule has a circular shape and possesses D9h symmetry. When the electric field strength increases to 0.020 a.u. (about 1 V/Å) or higher, B_9_N_9_ will clearly change to an elliptical shape, and the symmetry becomes Cs. B_9_N_9_ is treated as completely planar throughout the calculation.

The LBO is a tool for assessing the nature of chemical bonds. It is defined as the proportional integral of the negative part of the electron density Laplace equation in the fuzzy overlap space, with the expression:

(1) $$LBO_{A,B}=-10\times \underset{\nabla^{2}\rho <0}{\int }w_{A}\left(r\right)w_{B}\left(r\right)\nabla^{2}\rho \left(r\right)dr$$

In the fuzzy overlap space, the integral of the negative part of $\nabla^{2}\rho$, −10 is the empirical prefactor, *w*(*r*) denotes the weighting function, and *LBO* has a better ability to distinguish the bond strength. Figure 2 shows the variation in *LBO* of B_9_N_9_ at different EEF intensities. The EEFs corresponding to (a)–(f) are 0 a.u., 0.01 a.u., 0.02 a.u., 0.025 a.u., 0.03 a.u., and 0.0365 a.u., respectively. As we can see from (a), the bond level of B_9_N_9_ remains stable when no electric field is applied. From (b), the application of a smaller EEF (0.01 a.u.) shows a weak change in *LBO*, and with the increase in the electric field (c–f), the change in *LBO* will be more significant due to the redistribution of the charge inside the molecule caused by the application of the electric field, which destabilizes the molecule. At EEF = 0.0365 a.u, the bond level of #14 decreases to 0.741 (f). At this point, if the EEF continues to increase, it will lead to atomic detachment and cause dissociative isomerization within the molecule. Therefore, the maximum EEF considered in this paper is 0.0365 a.u. Apparently, the EEF significantly affects the stability of B_9_N_9_ because of the redistribution of charge inside the molecule caused by the application of the electric field [18], which leads to dissociative isomerization inside the molecule.

Additionally, the effect of EEF on the geometry of cyclo[18]carbon is obvious. Lu et al. [31] reported the variation in *LBO* of cyclo[18]carbon under EEF. When EEF = 0, the *LBO* of cyclo[18]carbon alternated between 2.45 and 1.69, and when EEF = 0.029 a.u., the *LBO* changed from a maximum of 2.45 (EEF = 0 a.u.) to 1.35 (EEF= 0.029 a.u.) with a relative change of 44.89%, while the *LBO* of B_9_N_9_ decreased from 1.18 (EEF = 0 a.u.) to 0.95 (EEF = 0.029 a.u.) with a relative change of 19.49%; thus, it can be concluded that the application of the same intensity of EEF causes a more significant change in cyclo[18]carbon compared to B_9_N_9_, indicating that B_9_N_9_ has relatively high stability.

We also made a choice of the direction of the applied electric field by using the *LBO* tool. When EEF = 0.0365 a.u., there is a significant distortion of B_9_N_9_ in the z-direction and the molecules are not in the same plane with a minimum *LBO* = 1.03, and the minimum *LBO* = 0.74 for the lower right corner of the molecule B-N in the y-direction at this time. In addition, the minimum *LBO* is 0.81 when the electric field is applied in the x-direction. Therefore, it can be inferred that the application of electric field in the y-direction causes the B-N bond in the lower right corner of B_9_N_9_ to break at smaller electric field strength.

**Figure 3:**
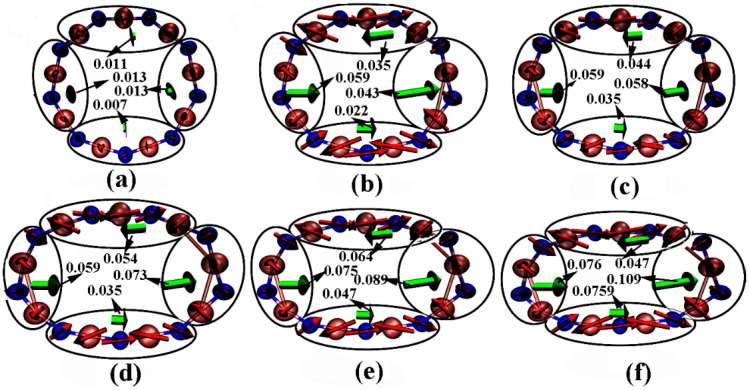
Diagram of B_9_N_9_ molecules under varying EEF strengths. The red arrows represent the atomic force; the length of each arrow represents the magnitude of the force. In each figure, two green arrows on each side represent the resultant forces (in Hartree/Bohr) of the atoms within each circled region of the molecule. The isovalue is chosen to be 0.03. The EEFs corresponding to (**a**–**f**) are 0.005 a.u., 0.015 a.u., 0.02 a.u., 0.025 a.u., 0.03 a.u., and 0.0365 a.u., respectively.

The primary explanation for the obvious change in the geometric structure of the B_9_N_9_ molecule is the vector sum of the atomic forces. To illustrate this concept, force diagrams of the B_9_N_9_ molecule under varying EEF strengths are drawn in Figure 3, focusing on the atomic force f [31], given by the equation *f* = qE, where q is the charge on each atom, and E is the electric field vector. Because the external electric field inevitably leads to a redistribution of the molecular charge, the electric field distribution inside the molecule is no longer uniform, resulting in molecular polarization, causing a reorganization of the bond lengths and bond angles inside the molecule. To illustrate, we use a red arrow to mark the atomic force on each atom. The four regions inside B_9_N_9_ that form the combined force are marked with green arrows, the length of which describes the magnitude of the force. As can be seen from Figure 3, under the electric field effect, most of the atoms close to the electric field source are negatively charged, while most of the atoms far from the source are positively charged. As the electric field increases, molecular polarization becomes increasingly pronounced. The increasing force inside the molecule further causes B_9_N_9_ to approach an elliptical structure.

### 3.2. Polarization Properties of B_9_N_9_ under an EEF

**Figure 4:**
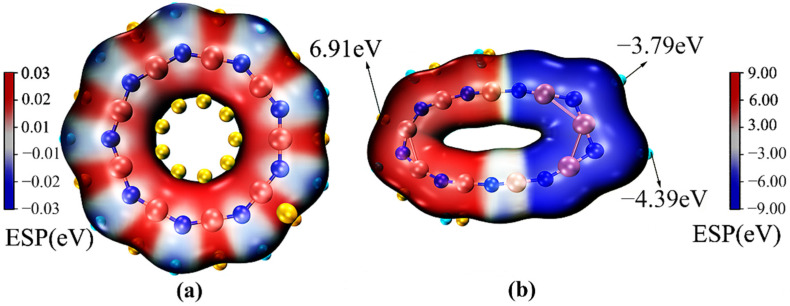
Distribution of the electric potential on the van der Waals surface of the B_9_N_9_ molecule; (**a**) depicts the ESP in the absence of an EEF; (**b**) depicts the ESP at EEF = 0.0365 a.u.

To study the electronic structure of the B_9_N_9_ molecule in the presence of an external electric field, we applied electrostatic potential (ESP) [32] analysis to the van der Waals surface [33]. Figure 4a,b show the distribution of the ESP on the van der Waals surface of the B_9_N_9_ molecule when there is no electric field and when EEF = 0.0365 a.u., respectively. The green balls and the yellow balls on the molecular surfaces represent the extreme negative point, corresponding to the B atoms, and the extreme positive point, corresponding to the N atoms. When there is no electric field, the electrostatic potential distribution on the surface of B_9_N_9_ attributable to van der Waals forces is relatively small—between −1.5 and 1.2 eV—because the internal molecular action is provided mainly by exchange repulsion and dispersion attraction, and this composite interaction is relatively weak. When the external electric field strength is 0.0365 a.u., the distribution range of the electrostatic potential on the surface of B_9_N_9_ is expanded and the polarization of the electron density is significant: the electrostatic potential on the side of the molecule closer to the source of the electric field is negative, reaching −4.385 eV, whereas the potential on the far side is positive—the maximum can reach 6.91 eV.

In addition, we included the molecular polarity index *MPI* [33] in calculating the molecular polarity of B_9_N_9_ at varying EEF strengths. The strength of the weak interaction can be well predicted by analysis of the ESP on vdW surfaces by analyzing the magnitude and position of the electrostatic potential on the van der Waals (vdW) surface, and the *MPI* is a descriptor of molecular polarity measured by the molecular surface electrostatic potential (ESP). The average of ESP over the entire surface is:

(2) VS=(1t)∑k=1tV(rk)

The *MPI* is closely related to the ${\bar{V}}_{S}$ index:

(3) MPI=(1t)|∑k=1tV(rk)|≡(1A)∬S|V(r)|dS

where *A* and *V*(*r*) are the area of the van der Waals surface and the value of the ESP at point *r* in space, respectively. It can be seen from Table 2 that the *MPI* is able to quantify the local polarity induced by the characteristic inhomogeneity of the ESP distribution. The calculated *MPI* value of 0.45739168 eV is obtained in the absence of an electric field. The nonpolarized and polarized areas are 50.91% and 49.09%, respectively, which shows that the polarity of the B_9_N_9_ molecule is quite low. A comparison of the *MPI* values of B_9_N_9_ molecules under different electric fields shows that with increasing external electric field strength, the *MPI* values of B_9_N_9_ molecules increase, the percentage of the area that is polar increases, and the percentage of the area that is nonpolar decreases. When the external electric field strength is 0.0365 a.u., the *MPI* value reaches 2.2346 eV and the polar area reaches 89.5%, showing that the molecule becomes highly polar. Therefore, an increase in the EEF intensity will transform the polarity of B_9_N_9_ from weak to strong, and its reactivity will be significantly enhanced.

For EEF = 0, the MPI value of cyclo[18]carbon is: 0.11234346 eV (2.6 kcal/mol) [33] and the percentage of nonpolar surface area is close to 100%, which reflects the obvious nonpolar behavior. The MPI value changed to 2.90178951 at EEF = 0.029 a.u. The polar surface area became larger, and the percentage of polar surface area reached 93.69%; thus, it can be inferred that a significant polarization occurred in cyclo[18]carbon. In contrast, the MPI of B_9_N_9_ changed from 0.45 (EEF = 0) to 1.71 (EEF = 0.029 a.u.), and the polarized area share changed from 49.09% to 85.19%. It is evident that both molecules undergo polarization under the action of EEF; nevertheless, the polarization of cyclo[18]carbon is more pronounced under the same EEF action, while B_9_N_9_ requires a greater electric field. The analysis of the energy of the two MOs shows that the HOMO–LUMO gap of cyclo[18]carbon is 6.75 eV, while the HOMO–LUMO gap of B_9_N_9_ is 10.3 eV; it is easier to be polarized by molecules with smaller energy gaps.

### 3.3. Effect of an EEF on Nonlinear Optical Properties of B_9_N_9_

Modulation with an external field facilitates the emergence of nonlinear optical (NLO) properties, [34] which, along with electric properties, depend on the influence of the external field intensity. As shown in Equation (4), the molecular polarization *α* and hyperpolarization *β* are the response characteristics of the molecule to the applied electric field, which leads to the polarization of the molecule. The change in polarization will eventually lead to a change in the dipole moment, whereas the hyperpolarization represents a nonlinear polarization effect. The relevant data were calculated by the coupled-reagent Kohn–Sham (CPKS) method [35] and are summarized in Table 3.

Therefore, the response of the molecular system to EEF is driven by the electron polarization, and the dipole moment is the most direct indicator of the degree of EEF polarization. The direct analytical differentiation of the EEF using the dipole moment is a common method to assess polarization rate at the theoretical level, and the induced dipole moment of the molecular system under the action of EEF can be written as:

(4) $$\mu =-\frac{\partial E}{\partial F}=\mu_{0}+\alpha F+\frac{1}{2}\beta F^{2}+\ldots$$

**Figure 5:**
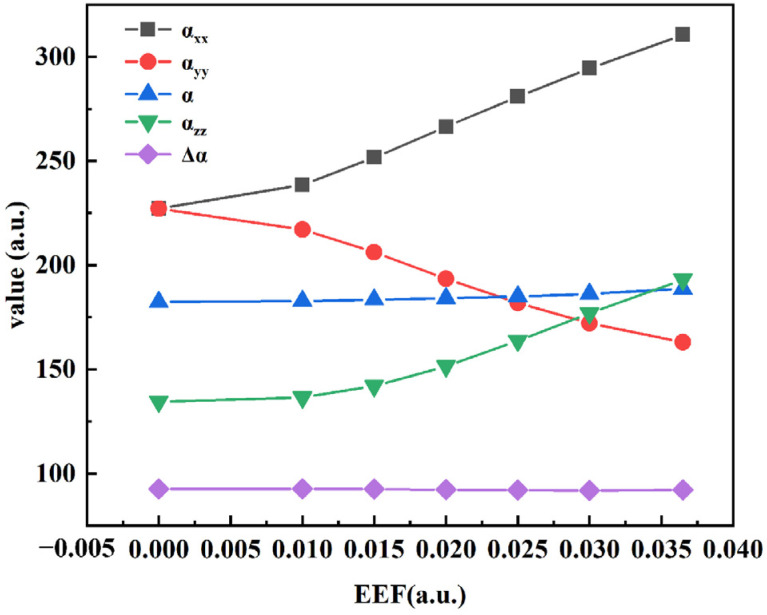
Properties of polarization at different EEF strengths.

Combining the information obtained from Figure 5 confirms that the dipole moment increases significantly with the increase in EEF strength, and that the molecular structure clearly changes. Additionally, the applied electric field causes molecular polarization, the polarization *α*, and the first hyperpolarization *β* of the molecule to change significantly. We will further examine the variation in these parameters with the external electric field strength. The polarizations (*α*_xx_ and *α*_yy_) on the circular plane parallel to B_9_N_9_ are significantly more active than those perpendicular to the plane (*α*_yy_) due to the large number of pi electrons leaving the domain.

Additionally, since the applied EEF is in the horizontal direction, the polarization is more active in the direction parallel to the B_9_N_9_ structure, although the polarization does not show sensitivity in the z-direction. Analyzing the trends in ∆*α* demonstrates that B_9_N_9_ has a significant polarization anisotropy, which leads to the polarized distribution of pi electrons with an increase in the external electric field strength, resulting in the generation of a stronger induced dipole moment. All data obtained from calculations of the polarization and the hyperpolarization at different EEF strengths are listed in Table 3.

**Figure 6:**
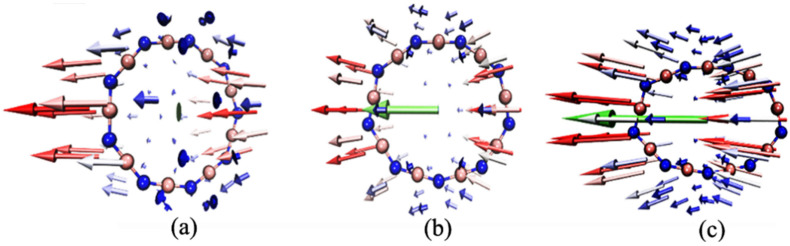
Visualization of the first hyperpolarization at different EEF strengths. (**a**) EEF = 0 a.u., (**b**) EEF = 0.015 a.u., (**c**) EEF = 0.025 a.u. The direction of the green arrow reflects the total trend (vector sum) of all arrows of the system, and its length reflects the intensity of the first hyperpolarization rate.

The first hyperpolarizability visualization images, displayed in Figure 6, were obtained using the Multiwfn3.8 program for three selected EEF intensities, from which we conclude that the first hyperpolarizability is incrementally consistent with the external electric field variation, i.e., it shows an obvious increasing trend in the direction of the electric field source as the electric field intensity increases.

### 3.4. Predicting the Change in Reactivity of B_9_N_9_ in an Applied EEF

The electrostatic potential distribution on the van der Waals surface of a molecule is a common method used to predict electrophilic and nucleophilic reaction sites [36]. The relevant description is given in Equation (5):

(5) $$V_{tot}\left(r\right)=V_{nuc}\left(r\right)+V_{ele}\left(r\right)=\underset{A}{\sum }\frac{Z_{A}}{r-R_{A}}-\int \frac{\rho \left(r^{\prime }\right)}{r-r^{\prime }}dr^{\prime }$$

where *Z* is the nuclear charge of atom, *A*, *R* is the nucleus coordinate, and ρ is the electron density. From Equation (5), it can be seen that the electrostatic potential consists of two components: nuclear charge and electron density. The more positive electrostatic potential means that nucleophilic reactions are more likely to occur in this region, and the more negative electrostatic potential means that electrophilic reactions are more likely to occur in this region. Figure 4 projects the electrostatic potential onto the surface of the molecule in different colors according to the value, with the red area reflecting positive charge and the blue area reflecting negative charge. The potential of B_9_N_9_ molecules in Figure 4a is uniformly distributed when the electric field is not added, and the extreme points of the electrostatic potential appear alternately on the circle, which indicates that the probability of producing electrophilic and nucleophilic reactions of B_9_N_9_ is the same when EEF = 0. In Figure 4b, due to the redistribution of electrostatic potential caused by EEF, when EEF = 0.0365 a.u., the left side of the molecule shows electropositivity and is more susceptible to nucleophilic attack; the right side shows electronegativity and is more susceptible to electrophilic attack.

An applied EEF leads to a significant change in B_9_N_9_ geometry and electronic properties and also affects the tendency for the substitution reactions of B_9_N_9_ to be preferentially nucleophilic or electrophilic. Therefore, predicting the active sites of electrophilic substitution reactions is of great theoretical and practical importance. The Fukui function [37] (FF) and the orbital weight double descriptor [38] (DD) based on Fukui function theory are widely used for the prediction of active sites for electrophilic substitution reactions, as calculated in Equation (6):

(6) $$f\left(r\right)={\left[\frac{\partial \mu }{\partial \nu \left(r\right)}\right]}_{N}={\left[\frac{\partial \rho \left(r\right)}{\partial N)}\right]}_{\nu \left(r\right)}$$

where *N* represents the number of electrons in the current system, *µ* is the system chemical potential, and *v*(*r*) represents the attraction potential of the nucleus for electrons. The partial derivative of the electron density with respect to *N*, determined by the geometry of the system, is discontinuous when *N* is an integer. The expression for an electrophilic reaction is given in Equation (7):

(7) $$f_{w}^{+}\left(r\right)=\overset{\infty }{\underset{i=LUMO}{\sum }}w_{i}{\left|\varphi_{i}\left(r\right)\right|}^{2}$$

(8) $$f^{-}\left(r\right)=\rho_{N}\left(r\right)-\rho_{N-1}\left(r\right)\approx \rho^{HOMO}\left(r\right)$$

(9) $$f^{+}\left(r\right)=\rho_{N1+}\left(r\right)-\rho_{N}\left(r\right)\approx \rho^{LUMO}\left(r\right)$$

(10) $$f^{2}\left(r\right)=f^{+}\left(r\right)-f^{-}\left(r\right)\approx \rho^{LUMO}\left(r\right)-\rho^{HOMO}\left(r\right)$$

**Figure 7:**
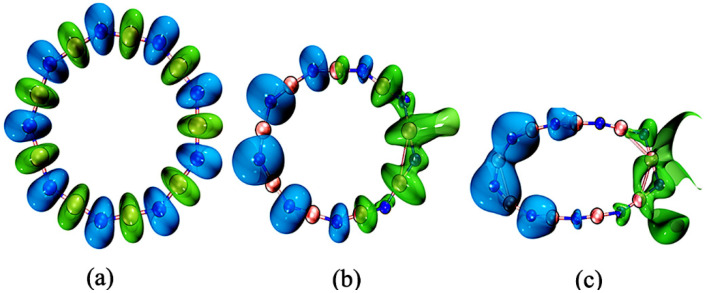
Visualization of dual-descriptor-predicted reactive sites at different EEF strengths; (**a**–**c**) are dual descriptor isograms; The isovalue is chosen to be 0.03.

The *ρ_N_*(*r*), *ρ*_*N*1+_ (*r*), and *ρ*_*N*−1_(*r*) represent the electron densities of the system in the original state (*N* electrons), combined one-electron state (*N*1+ electrons), and ionized one-electron state (*N* − 1 electrons), respectively. From Equation (8), ƒ^−^ shows the degree of change in electron density at each position when the system is subject to charge transfer due to electrophilic attack, and ƒ^+^ in Equation (9) shows the nucleophilic reactivity of different regions. The ƒ^2^(*r*) in Equation (10) represents the expression of the double description, which is used here to predict the visualized images of electrophilic and nucleophilic reactive sites of B_9_N_9_ under the action of EEFs of different intensities (0 a.u., 0.015 a.u., and 0.0365 a.u.). This method was used to predict the electrophilic and nucleophilic responses of B_9_N_9_ under the action of EEF at different intensities (0 a.u., 0.015 a.u., and 0.0365 a.u.). In Figure 7a–c, the blue color represents sites prone to electrophilic reactions and the green color represents sites prone to nucleophilic reactions. 

The reactivity of B_9_N_9_ showed significant stress with the increase in EEF strength. Equation (10) implies that B_9_N_9_ is more susceptible to nucleophilic attack in the region for which ƒ^2^(*r*) < 0, whereas ƒ^2^(*r*) > 0 describes the condition under which electrophilic attack is more favorable. 

According to the analysis in Figure 7, when EEF = 0.0365 a.u., the electrophilic reaction is more likely to occur on the left side of B_9_N_9_ and the nucleophilic reaction is more likely to occur on the right side of the molecule.

**Figure 8:**
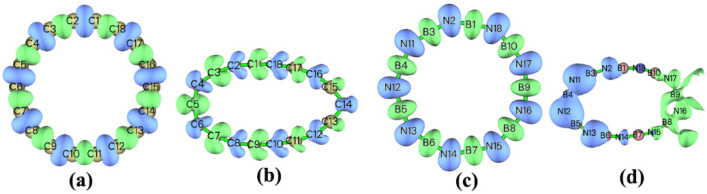
Comparison of the reactivity sites predicted by the double descriptors for cyclo[18]carbon and B_9_N_9_ at the same EEF at different EEF intensities; (**a**,**b**) for cyclo[18]carbon, corresponding to an EEF of 0.029 a.u. and (**c**,**d**) for B_9_N_9_, corresponding to an EEF of 0.029 a.u., respectively.

We have also analyzed the variation in the double descriptors of cyclo[18]carbon under different strengths of electric fields. As can be seen from Figure 8a, when EEF = 0, the electrophilic reaction easily occurs in short C-C bonds, and the nucleophilic reaction [39] is more likely to occur in long C-C bonds. As can be seen from Figure 8b, when EEF = 0.029 a.u., the surface of the cyclo[18]carbon ring reflects obvious alternating sites for electrophilic and nucleophilic reactions. As shown in Figure 8c, when EEF = 0, the distribution of B_9_N_9_ reaction sites is relatively uniform, and it can be seen from Figure 8d that when EEF = 0.029 a.u., the surface of B_9_N_9_ shows a clear separation of electrophilic and nucleophilic reaction regions. This is due to the re-separation of electrons on the surface of the molecule as the electric field increases; however, a greater separation of electrons from the domain appears on the surface of B_9_N_9_ than that of cyclo[18]carbon. 

### 3.5. Excitation Effect of an Applied EEF on B_9_N_9_

**Figure 9:**
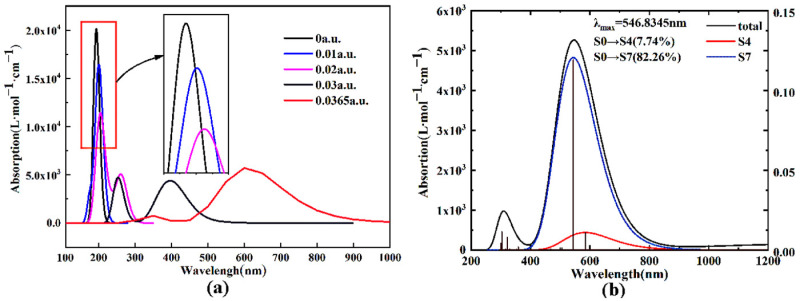
Modulation effect of external electric fields on the absorption spectrum of B_9_N_9_; (**a**) the total absorption spectrum curve; (**b**) the contribution curves from the electronic excitations with vibrational intensity greater than 0.01.

The excitation energy and the vibronic intensity electronic absorption spectra of the excited states of B_9_N_9_ at different EEF intensities were calculated using time-dependent density functional theory (TD-DFT), and the electronic absorption spectra were plotted in Figure 9. Compared to observations when no field is present, the spectral characteristics did not change much when the EEF was in the range of 0 to 0.01 a.u. There is no absorption in the visible region, but there is a very strong absorption peak in the UV region near 200 nm. As the EEF strength increased, starting at around 0.02 a.u., the absorption in the UV region gradually diminished, and a distinct new absorption band appeared in the visible region, indicating that the EEF has a significant modulating effect on the absorption spectrum of B_9_N_9_. In the TD-DFT calculations, a portion of the electron excitation arising from the jump from occupied to unoccupied orbitals leads to a weakening of the absorption spectrum intensity near 200 nm.

**Figure 10:**
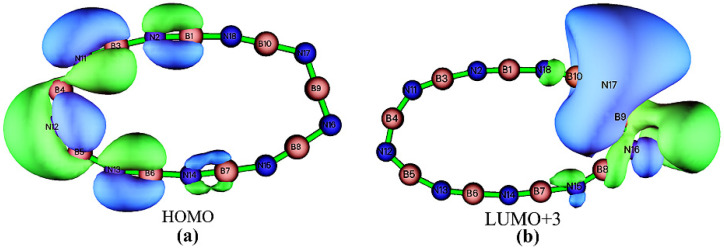
Isosurface maps of the (**a**) HOMO and (**b**) LUMO + 3 of B_9_N_9_ corresponding to the application of EEF = 0.0365 a.u. The isovalue is chosen to be 0.03. The positive and negative orbital phases are distinguished by the two colors.

The mechanism of the new absorption band induced by the EEF in the visible region will be explored below. Here, we take EEF = 0.0365 a.u. as an example for our analysis. Firstly, the total absorption spectrum curve in Figure 9b is decomposed, and the contribution curves from the electronic excitations with vibrational intensity greater than 0.01 are plotted separately in Figure 9a. The maximum absorption band generated by the EEF at 546.8345 nm is mainly caused by the S0→S7 excitation, to which the HOMO-1 to LUMO + 3 jump is the main contributor. Therefore, we further plotted the contours of the relevant orbitals to study their properties, as visualized in Figure 10. The HOMO-1 is a π orbital that has been partially polarized, while the LUMO + 3 is a π∗ orbital. In the S0→S7 excitation process, two different orbital jumps, π→π∗, are mainly responsible for the appearance of a distinct absorption band in the visible region. In addition, the intensity of the absorption peak decreases due to the molecular polarization effect.

### 3.6. The Effects of Cations on the Molecular Structure of B_9_N_9_

**Figure 11:**
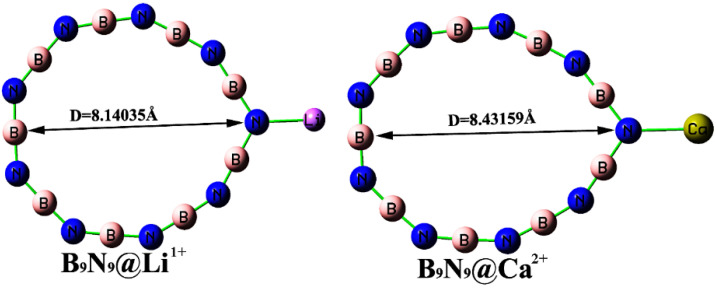
Structure of the two complexes.

The study of the effect on molecular structure by introducing cations [31] is a novel idea, which may be more conducive to the exploration of molecular properties by EEF. We introduced three monovalent alkali metal ions (Li^1+^, Na^1+^, K^1+^) and three divalent alkaline earth metal ions (Be^2+^, Mg^2+^, Ca^2+^) to form complexes with B_9_N_9_, and investigated the changes in B_9_N_9_ properties under the action of different ions. Table 4 depicts the geometric structure and charge transfer of different ions for B_9_N_9_. Combined with Figure 11, it is clear that the geometric change and charge transfer of Li ions for B_9_N_9_ are obvious in the presence of Li^1+^. It is also obvious that the induction of divalent ions is more significant than that of monovalent ions for the B_9_N_9_ molecule; we can also observe that, regarding the geometric change in Li ions for B_9_N_9_@Li^1+^, B_9_N_9_@Na^1+^, and B_9_N_9_@K^1+^ complexes, the geometric change in D of B_9_N_9_@Li^1+^ relative to that of B_9_N_9_ is the largest (0.68475e), which is due to the fact that Li^1+^ has the smallest radius and the largest charge density, meaning that more electrons transfer from B_9_N_9_ to the ion, therefore creating a greater effect on the geometry of B_9_N_9_. The effect of divalent ions on the B_9_N_9_ molecule also obeys this principle. This is in agreement with the report of Lu et al.

**Figure 12:**
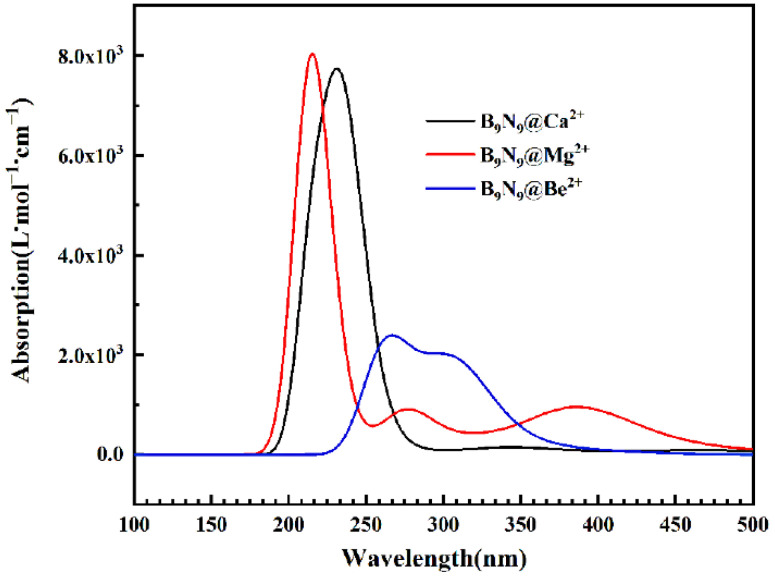
Absorption spectra of Be^2+^, Mg^2+^, and Ca^2+^ for B_9_N_9_.

We also studied the excited states of the B_9_N_9_ ion complex. Figure 12 depicts the absorption spectrum of the divalent ion for B_9_N_9_, and it can be seen that a new absorption peak (277.25 nm) appears in the absorption spectrum of B_9_N_9_@Mg^2+^, which is caused by S0–S8 excitation. We can also observe that the absorption peak of the absorption spectrum of B9N9@Be^2+^ (blue curve) is located at 263.38 nm. The absorption spectrum is red-shifted and the absorption intensity is weakened; the absorption peak is caused by S0→S10 excitation, which is due to the severe polarization of B_9_N_9_ as increasing numbers of electrons are transferred on the molecule and increasing orbitals break the prohibition and cause more and more orbital jumps, thus weakening the absorption intensity. We can assume that the introduction of charged ions in the vicinity of the molecule produces a similar effect to that of EEF.

## 4. Conclusions

In this paper, theoretical calculations established that the cyclic structure of B_9_N_9_ is severely deformed from circular to elliptical and that the Laplacian bond order is significantly weakened as the EEF strength increases. The action of the EEF inside the molecule causes changes in the electronic structure of the system: the polarization of the molecule increases, the nonlinear optical effect manifests, and the polarization increases. The application of an EEF was found to alter the reactivity of B_9_N_9_ significantly after calculations involving double descriptors, and the Fukui function predicted the reactive site of the molecule, which determines whether a substitution reaction will tend to be electrophilic or nucleophilic based on shifts in polarity. Excited state calculations found that the presence of an EEF significantly improved the electronic spectral absorption of B_9_N_9_. When the EEF strength exceeded roughly 0.02 a.u., the molecular orbital excitations became active with increasing EEF strength, weakening the intensity of the very strong absorption peak in the UV region and the appearance of a significant absorption band in the visible region due to the active manifestation of π→π∗ orbital jumps. The addition of alkali metal and alkaline earth metal ions in the vicinity of the B_9_N_9_ molecule allows for the modulation of molecular geometry and absorption spectra. The understanding of the effects of an applied EEF that we have gained through this research will contribute greatly to improving the catalytic performance of B_9_N_9_, and should expand the applications of this material.

## Figures and Tables

**Table 1:** Structural changes of B_9_N_9_ under different computational conditions.

Method	Basis Set	R(Å)	∠NBN°	∠BNB°
ωB97XD	def2-TZVP	1.312	140.7	179.3
CAMB3LYP [14]	6–311G(d)	1.311	140.9	179.1

**Table 2:** Parameters such as MPI index, unpolarized area, and polarized area under different electric fields.

EEF (a.u.)	Molecular Polarity Index MPI (eV)	Overall Surface Area (Angstrom^2^)	Nonpolar Surface (Angstrom^2^)	Polar Surface (Angstrom^2^)	Percentage of Nonpolar Surface Area (%)	Percentage of Polar Surface Area (%)
0	0.45739168	282.65831	143.89	138.77	50.91	49.09
0.005	0.51278426	282.77317	132.12	150.66	46.72	53.28
0.01	0.68570757	283.04897	93.48	189.57	33.03	66.97
0.015	0.91479659	273.74361	64.01	219.44	22.58	77.42
0.02	1.17538361	283.90786	54.48	229.43	19.19	80.81
0.025	1.46101180	284.43305	51.16	233.27	17.99	82.01
0.03	1.77809747	284.86773	39.65	245.21	13.92	86.08
0.0365	2.23461782	284.94954	29.93	255.02	10.5	89.5

**Table 3:** Various polarization data under different external electric fields (EEF).

EEF (a.u.)	0	0.01	0.015	0.02	0.025	0.03	0.0365
Μ (a.u.)	0.00039	0.89651	1.403169	1.950982	2.529925	3.123179	3.874655
*α*_xx_ (a.u.)	227.147	238.555	251.643	266.415	280.933	294.5	310.634
*α*_yy_ (a.u.)	227.097	217.098	206.218	193.497	181.904	172.14	163.052
*α*_zz_ (a.u.)	92.6169	92.6039	92.518	92.2848	92.0281	91.9171	92.0991
*α* (a.u.)	182.287	182.7523	183.4597	184.0656	184.955	186.1857	188.595
∆*α* (a.u.)	134.5051	136.4934	141.9717	151.4634	163.6976	176.8549	193.0962
*β* (a.u.)	0.20429	282.0924	460.4275	666.9742	901.5195	1156.972	1517.852
*β*||(a.u.)	−0.12247	−169.255	−276.255	−400.184	−540.908	−694.177	−910.696

**Table 4:** Geometric structure and charge variation in different ionic complexes of B_9_N_9_.

	D(Å)	ΔD(Å)	Q(e)	ΔQ(e)
B_9_N_9_@K^1+^	7.94699	0.49139	0.940	−0.06
B_9_N_9_@Na^1+^	8.03905	0.58345	0.936	−0.064
B_9_N_9_@Li^1+^	8.14035	0.68475	0.908	−0.092
B_9_N_9_@Ca^2+^	8.43159	0.97599	1.709	−0.291
B_9_N_9_@Mg^2+^	8.51549	1.05989	1.349	−0.651
B_9_N_9_@Be^2+^	8.64304	1.18744	0.702	−1.298

1. D stands for the longest distance between two atoms on the B_9_N_9_ complex ring (as in Figure 12); ΔD is the difference between the longest distance on the B_9_N_9_ complex and the longest distance on B_9_N_9_ (Figure 1). 2. Q is representative of the charge carried by the ion on the B_9_N_9_ complex, and ΔQ is representative of the charge transferred from the electron to the ion on B_9_N_9_.

## Data Availability

The authors confirm that the data supporting the findings of this study are available within the article and Supplementary Materials.

## Supplementary Materials

Table S1: LBO values at different EEF strengths; Table S2: Four regions of combined force data formed within B_9_N_9_ at different intensities of EEF; Table S3: Data of Fukui function and double descriptor; Table S4: Cartesian coordinates of B_9_N_9_ under different external electric field; Figure S1: The bond order number of B_9_N_9_; Figure S2: The division of the atomic force region of B_9_N_9_; Figure S3: Changes in the HOMO energy and LUMO energy of B_9_N_9_ under the action of an external electric field. The green generation is LUMO, and the red is HOMO.

## Sample Availability

Samples of the compounds are not available from the authors.
