# Supplementary material for: Model of B9N9 Response under External Electric Field: Geometry, Electronic Properties, Reaction Activity
Source: Molecules. 2022 Mar 6;27(5):1714. doi: 10.3390/molecules27051714 (PMC8912050; doi:10.3390/molecules27051714)
Supplement: Supplementary file 1 [file molecules-27-01714-s001.zip › molecules-1571038-supplementary.pdf]

Supplemental material of

Model of B_9N_9 response under external electric field: geometry, electronic properties, reaction activity

Xupu Wu[1,2] Dasen Ren[1,*]
[1]School of Mechatronics Engineering, Guizhou Minzu University, Guiyang, Guizhou, 550025, China
[2]School of Physics and Electronic Sciences, Guizhou Education University, Guiyang, Guizhou, 550018, China
*Correspondence author .Email: dasenren@sina.com

1 . Laplacian bond order (LBO) values at different EEF strengths and the Bond order number of B_9N_9.

Table S1 LBO values at different EEF strengths

Bond order number	0a.u.	0.015a.u.	0.02a.u.	0.025a.u.	0.03a.u.	0.0365a.u.
**1**	1.18161	1.26719	1.34532	1.37701	1.40304	1.4254
**2**	1.18071	1.10177	1.0184	0.97289	0.92382	0.85733
**3**	1.18179	1.13152	1.06135	1.02631	0.98263	0.92333
**4**	1.18161	1.24854	1.31100	1.34688	1.37807	1.40436
**5**	1.18034	1.16902	1.14637	1.1267	1.11191	1.0816
**6**	1.18023	1.19899	1.20185	1.20019	1.19524	1.19149
**7**	1.18159	1.22619	1.26183	1.27256	1.28562	1.30618
**8**	1.18196	1.15217	1.09909	1.0752	1.04021	0.98283
**9**	1.18078	1.26005	1.3393	1.37136	1.39941	1.42798
**10**	1.18198	1.10795	1.04175	0.99633	0.94736	0.88325
**11**	1.18172	1.25814	1.33635	1.36685	1.39295	1.41679
**12**	1.18015	1.09031	0.9946	0.94805	0.90089	0.83958
**13**	1.18109	1.21794	1.26513	1.2916	1.3191	1.34097
**14**	1.18289	1.11588	0.99823	0.9278	0.8506	0.74095
**15**	1.18287	1.14188	1.08057	1.05072	1.0158	0.97274
**16**	1.18114	1.18127	1.18146	1.18428	1.1938	1.21489
**17**	1.18021	1.09913	0.97259	0.90767	0.84145	0.75839
**18**	1.18041	1.24561	1.3144	1.34278	1.3646	1.38351

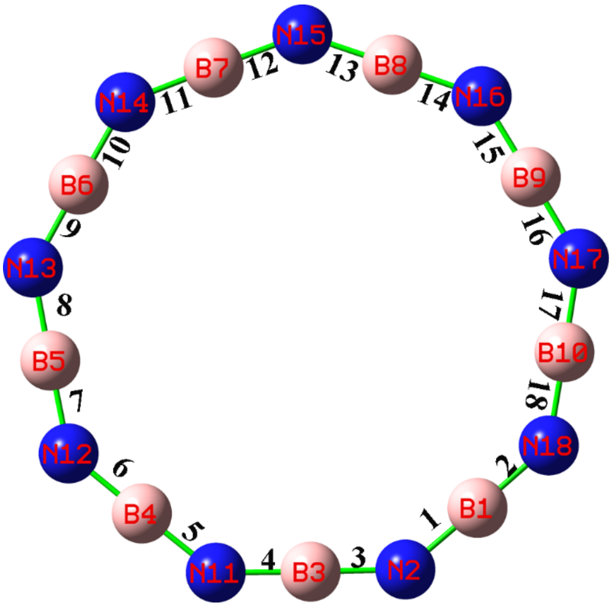

Figure S1 The Bond order number of B_9N_9

2 Four regions of combined force data formed within B_9N_9 at different intensities of EEF and

TableS2 Four regions of combined force(in Hartree/Bohr) data formed within B9N9 at different intensities of EEF

Region Number	1	2	3	4
EEF=0.005a.u.	0.013	0.011	0.013	0.007
EEF=0.015a.u.	0.059	0.035	0.043	0.022
EEF=0.02a.u.	0.059	0.044	0.058	0.035
EEF=0.025a.u.	0.059	0.054	0.073	0.035
EEF=0.03a.u.	0.075	0.064	0.089	0.047
EEF=0.035a.u.	0.076	0.047	0.109	0.0759

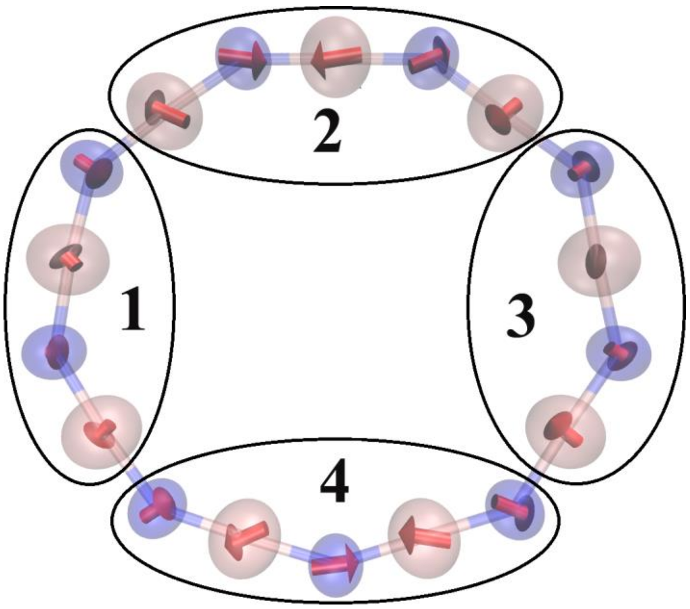

Figure S2 The division of the atomic force region of B_9N_9

3 Data of Fukui function and double descriptor

Table S3-1 Data of Fukui function and double descriptor when EEF = 0a.u.

Atom index	OW f+	OW f-	OW f0	OW DD
1(B)	0.0823	0.02673	0.05451	0.05557
2(N)	0.02878	0.08437	0.05657	-0.05559
3(B)	0.08229	0.02672	0.05451	0.05557
4(B)	0.08227	0.02672	0.05449	0.05555
5(B)	0.08229	0.02672	0.0545	0.05557
6(B)	0.08231	0.02672	0.05452	0.05559
7(B)	0.08227	0.02673	0.0545	0.05555
8(B)	0.08227	0.02672	0.05449	0.05555
9(B)	0.0823	0.02671	0.05451	0.05558
10(B)	0.08229	0.02672	0.05451	0.05557
11(N)	0.02877	0.08437	0.05657	-0.0556
12(N)	0.02877	0.08438	0.05658	-0.05561
13(N)	0.02878	0.08437	0.05657	-0.05559
14(N)	0.02878	0.08437	0.05658	-0.0556
15(N)	0.02877	0.08438	0.05657	-0.0556
16(N)	0.02878	0.08437	0.05657	-0.05559
17(N)	0.02878	0.08438	0.05658	-0.0556
18(N)	0.02878	0.08439	0.05658	-0.05561

[1]OW f- shows the degree of electrophilic reaction
[2]OW f+ shows the degree of electrophilic reaction
[3]OW f0 shows the degree of free radical reaction
[4]OW DD the regions with negative values are more likely to be electrophilic sites, and the more regular values are, the more vulnerable they are to nucleophilic attack

Table S3-2 Data of Fukui function and double descriptor when EEF = 0.005a.u.

Atom index	OW f+	OW f-	OW f0	OW DD
1(B)	0.08438	0.02432	0.05435	0.06007
2(N)	0.02509	0.08636	0.05572	-0.06126
3(B)	0.05846	0.03326	0.04586	0.0252
4(B)	0.04522	0.03951	0.04237	0.00571
5(B)	0.04522	0.03951	0.04237	0.00571
6(B)	0.05846	0.03326	0.04586	0.0252
7(B)	0.08438	0.02432	0.05435	0.06007
8(B)	0.11543	0.01737	0.0664	0.09806
9(B)	0.13084	0.0149	0.07287	0.11594
10(B)	0.11543	0.01737	0.0664	0.09806
11(N)	0.01794	0.12021	0.06907	-0.10227
12(N)	0.01548	0.13674	0.07611	-0.12126
13(N)	0.01794	0.12021	0.06907	-0.10227
14(N)	0.02509	0.08636	0.05572	-0.06126
15(N)	0.03558	0.05844	0.04701	-0.02286
16(N)	0.04454	0.04464	0.04459	-0.0001
17(N)	0.04454	0.04464	0.04459	-0.0001
18(N)	0.03558	0.05844	0.04701	-0.02286

Table S3-3 Data of Fukui function and double descriptor when EEF = 0.015a.u.

Atom index	OW f+	OW f-	OW f0	OW DD
1(B)	0.06291	0.02588	0.04439	0.03703
2(N)	0.01687	0.0923	0.05458	-0.07544
3(B)	0.03067	0.04296	0.03682	-0.01229
4(B)	0.01943	0.05224	0.03584	-0.03281
5(B)	0.02291	0.04921	0.03606	-0.0263
6(B)	0.04335	0.03472	0.03903	0.00863
7(B)	0.08928	0.01844	0.05386	0.07083
8(B)	0.15751	0.0092	0.08336	0.14832
9(B)	0.182	0.00747	0.09473	0.17453
10(B)	0.123	0.01274	0.06787	0.11026
11(N)	0.00882	0.16317	0.086	-0.15436
12(N)	0.00729	0.18622	0.09675	-0.17893
13(N)	0.01189	0.12837	0.07013	-0.11648
14(N)	0.02376	0.06367	0.04371	-0.03991
15(N)	0.04469	0.02979	0.03724	0.01489
16(N)	0.06491	0.01876	0.04184	0.04615
17(N)	0.05685	0.02212	0.03949	0.03473
18(N)	0.03323	0.04283	0.03803	-0.00959

Table S3-4 Data of Fukui function and double descriptor when EEF = 0.02a.u..

Atom index	OW f+	OW f-	OW f0	OW DD
1(B)	0.04421	0.02496	0.03459	0.01925
2(N)	0.0113	0.0811	0.0462	-0.0698
3(B)	0.01506	0.04935	0.03221	-0.03429
4(B)	0.00705	0.06138	0.03422	-0.05433
5(B)	0.00936	0.05804	0.0337	-0.04867
6(B)	0.02536	0.03736	0.03136	-0.012
7(B)	0.074	0.0156	0.0448	0.0584
8(B)	0.18473	0.00534	0.09503	0.17938
9(B)	0.24191	0.00367	0.12279	0.23824
10(B)	0.12169	0.00901	0.06535	0.11268
11(N)	0.00426	0.18655	0.09541	-0.1823
12(N)	0.00301	0.22817	0.11559	-0.22516
13(N)	0.00681	0.13096	0.06889	-0.12416
14(N)	0.01808	0.04801	0.03304	-0.02993
15(N)	0.04618	0.01591	0.03104	0.03027
16(N)	0.08759	0.00761	0.0476	0.07998
17(N)	0.06945	0.00999	0.03972	0.05946
18(N)	0.02923	0.02707	0.02815	0.00217

Table S3-5 Data of Fukui function and double descriptor when EEF = 0.025a.u..

Atom index	OW f+	OW f-	OW f0	OW DD
1(B)	0.02631	0.02382	0.02507	0.00249
2(N)	0.00672	0.06773	0.03723	-0.06101
3(B)	0.00633	0.05481	0.03057	-0.04848
4(B)	0.00217	0.06833	0.03525	-0.06616
5(B)	0.00328	0.06576	0.03452	-0.06249
6(B)	0.01249	0.03925	0.02587	-0.02676
7(B)	0.05243	0.0136	0.03302	0.03883
8(B)	0.19817	0.00347	0.10082	0.1947
9(B)	0.31077	0.002	0.15639	0.30878
10(B)	0.10536	0.00682	0.05609	0.09853
11(N)	0.00195	0.19861	0.10028	-0.19666
12(N)	0.00118	0.26028	0.13073	-0.2591
13(N)	0.00356	0.12547	0.06452	-0.12191
14(N)	0.01192	0.03567	0.0238	-0.02375
15(N)	0.04257	0.00893	0.02575	0.03364
16(N)	0.11254	0.00329	0.05792	0.10924
17(N)	0.0793	0.00482	0.04206	0.07448
18(N)	0.02223	0.01725	0.01974	0.00497

Table S3-6 Data of Fukui function and double descriptor when EEF = 0.03a.u.

Atom index	OW f+	OW f-	OW f0	OW DD
1(B)	0.01368	0.02306	0.01837	-0.00939
2(N)	0.00362	0.05707	0.03034	-0.05345
3(B)	0.00241	0.06022	0.03131	-0.05781
4(B)	0.00065	0.07425	0.03745	-0.07359
5(B)	0.00111	0.07256	0.03684	-0.07144
6(B)	0.00562	0.04031	0.02296	-0.03468
7(B)	0.03435	0.01207	0.02321	0.02228
8(B)	0.19895	0.00256	0.10076	0.19639
9(B)	0.37451	0.00129	0.1879	0.37322
10(B)	0.0827	0.00564	0.04417	0.07706
11(N)	0.00089	0.2041	0.10249	-0.20321
12(N)	0.00051	0.28214	0.14133	-0.28164
13(N)	0.00186	0.11648	0.05917	-0.11462
14(N)	0.0075	0.02671	0.0171	-0.01921
15(N)	0.03656	0.00539	0.02097	0.03117
16(N)	0.13475	0.00166	0.06821	0.13308
17(N)	0.08435	0.00268	0.04352	0.08167
18(N)	0.01506	0.01171	0.01339	0.00335

Table S3-7 Data of Fukui function and double descriptor when EEF = 0.0365a.u.

Atom index	OW f+	OW f-	OW f0	OW DD
1(B)	0.00338	0.0234	0.01339	-0.02002
2(N)	0.00134	0.04588	0.02361	-0.04454
3(B)	0.00051	0.07109	0.0358	-0.07057
4(B)	0.00026	0.08521	0.04274	-0.08495
5(B)	0.00039	0.08302	0.04171	-0.08262
6(B)	0.00139	0.04406	0.02272	-0.04267
7(B)	0.01487	0.01104	0.01295	0.00382
8(B)	0.19173	0.00232	0.09703	0.18941
9(B)	0.44578	0.00206	0.22392	0.44372
10(B)	0.04841	0.00504	0.02672	0.04337
11(N)	0.00091	0.2071	0.10401	-0.20619
12(N)	0.00108	0.28224	0.14166	-0.28116
13(N)	0.00101	0.10842	0.05471	-0.10741
14(N)	0.00379	0.01723	0.01051	-0.01344
15(N)	0.02615	0.00262	0.01438	0.02352
16(N)	0.16865	0.00106	0.08485	0.16759
17(N)	0.08226	0.00147	0.04187	0.08079
18(N)	0.00669	0.00668	0.00668	0

3.Optimized geometries

All geometries given below were optimized under ωB97XD/def2-TZVP level, no imaginary frequency can be found.

Table S4 Cartesian coordinates of B9N9 under defferent external electric field

Cartesian coordinates when the external electric field (EEF) is 0a.u. (unit: Å)

B	0.62561016	3.52170905	0.000000
N	-0.65964687	3.7344207	0.000000
B	-1.82301805	3.10829195	0.000000
B	-3.44567485	1.23592581	0.000000
B	-3.44567485	-1.23592626	0.000000
B	-1.82301801	-3.10829264	0.000000
B	0.62561011	-3.52170929	0.000000
B	2.78063438	-2.30556466	0.000000
B	3.64020883	0.00000033	0.000000
B	2.78063462	2.30556521	0.000000
N	-2.95232998	2.45613251	0.000000
N	-3.87848754	-0.00000018	0.000000
N	-2.95232977	-2.45613276	0.000000
N	-0.65964688	-3.73442135	0.000000
N	1.92927934	-3.29671872	0.000000
N	3.65218649	-1.31375802	0.000000
N	3.65218684	1.31375877	0.000000
N	1.92927957	3.2967194	0.000000

Cartesian coordinates when the external electric field (EEF) is 0.005a.u. (unit: Å)

B	0.00000000	3.28079868	0.000000
N	-1.28169626	3.26750537	0.000000
B	-2.44738041	2.61229261	0.000000
B	-3.91508092	0.6309502	0.000000
B	-3.3993477	-1.74230616	0.000000
B	-1.29904585	-3.11275949	0.000000
B	1.24569618	-3.11024437	0.000000
B	3.31561734	-1.73387661	0.000000
B	3.82954175	0.59708201	0.000000
B	2.42881328	2.57824661	0.000000
N	-3.51832431	1.89169047	0.000000
N	-4.12470962	-0.66309839	0.000000
N	-2.53162686	-2.7508918	0.000000
N	0.00395302	-3.43161982	0.000000
N	2.550194	-2.78164445	0.000000
N	4.15060984	-0.68909587	0.000000
N	3.57983242	1.88471845	0.000000
N	1.34404374	3.27230497	0.000000

Cartesian coordinates when the external electric field (EEF) is 0.020a.u. (unit: Å)

B	0.00000000	3.05508989	0.000000
N	-1.27244082	3.04508441	0.000000
B	-2.50537848	2.49989634	0.000000
B	-4.10643253	0.63393294	0.000000
B	-3.53897487	-1.68944852	0.000000
B	-1.32734692	-2.91510689	0.000000
B	1.25114446	-2.92393649	0.000000
B	3.43005439	-1.68922411	0.000000
B	3.9964715	0.57543022	0.000000
B	2.49163203	2.4520589	0.000000
N	-3.62469368	1.87080564	0.000000
N	-4.32675857	-0.65937485	0.000000
N	-2.56589056	-2.60988575	0.000000
N	0.00181121	-3.16856059	0.000000
N	2.5841527	-2.66479819	0.000000
N	4.35679046	-0.70758333	0.000000
N	3.71174193	1.85570905	0.000000
N	1.35588048	3.03953771	0.000000

Cartesian coordinates when the external electric field (EEF) is 0.025a.u. (unit: Å)

B	0.00000000	2.83058605	0.000000
N	-1.26424657	2.80644961	0.000000
B	-2.54789916	2.3546065	0.000000
B	-4.28072025	0.59871422	0.000000
B	-3.67268037	-1.67027119	0.000000
B	-1.35659907	-2.73238495	0.000000
B	1.24748463	-2.71367784	0.000000
B	3.53266501	-1.60664183	0.000000
B	4.15497545	0.58563233	0.000000
B	2.5513435	2.35192507	0.000000
N	-3.69701557	1.79867477	0.000000
N	-4.51978368	-0.6921059	0.000000
N	-2.60128236	-2.49124432	0.000000
N	-0.0020311	-2.91221795	0.000000
N	2.59959017	-2.48798287	0.000000
N	4.54843066	-0.69487656	0.000000
N	3.83261379	1.85714266	0.000000
N	1.36903198	2.81724028	0.000000

Cartesian coordinates when the external electric field (EEF) is 0.03a.u. (unit: Å)

B	0.00000000	2.61708748	0.000000
N	-1.25774537	2.58260367	0.000000
B	-2.58096585	2.21055931	0.000000
B	-4.43530955	0.56229864	0.000000
B	-3.78884547	-1.65558091	0.000000
B	-1.38403089	-2.55349771	0.000000
B	1.23999178	-2.50086148	0.000000
B	3.62575317	-1.52555092	0.000000
B	4.2960969	0.59124574	0.000000
B	2.59674915	2.25344507	0.000000
N	-3.75108461	1.71728976	0.000000
N	-4.68620104	-0.72763135	0.000000
N	-2.6327481	-2.37571711	0.000000
N	-0.00786874	-2.6680869	0.000000
N	2.60907149	-2.29684963	0.000000
N	4.72426586	-0.68677664	0.000000
N	3.92662801	1.84903436	0.000000
N	1.38322589	2.60674443	0.000000

Cartesian coordinates when the external electric field (EEF) is 0.03a.u. (unit: Å)

B	0.00000000	2.42658527	0.000000
N	-1.25324303	2.38572288	0.000000
B	-2.60814056	2.0790723	0.000000
B	-4.57038775	0.53346627	0.000000
B	-3.88578991	-1.64157732	0.000000
B	-1.40857088	-2.38808487	0.000000
B	1.23255359	-2.30362811	0.000000
B	3.70737426	-1.45987606	0.000000
B	4.41546843	0.58645618	0.000000
B	2.62991467	2.15852819	0.000000
N	-3.79400747	1.64059067	0.000000
N	-4.82259872	-0.75759556	0.000000
N	-2.65860571	-2.26276278	0.000000
N	-0.01373624	-2.44843597	0.000000
N	2.61764062	-2.11200256	0.000000
N	4.87692046	-0.6885797	0.000000
N	3.99760698	1.82815993	0.000000
N	1.39829323	2.42137321	0.000000

Cartesian coordinates when the external electric field (EEF) is 0.0365a.u. (unit: Å)

B	0.00000000	2.37526072	0.000000
N	-1.25245129	2.33282753	0.000000
B	-2.6154094	2.04407445	0.000000
B	-4.60814368	0.52720551	0.000000
B	-3.91109663	-1.63955318	0.000000
B	-1.41551242	-2.34210689	0.000000
B	1.23019974	-2.24707221	0.000000
B	3.72851885	-1.44192942	0.000000
B	4.45017447	0.58415536	0.000000
B	2.63767613	2.12983821	0.000000
N	-3.80480086	1.61869574	0.000000
N	-4.85610387	-0.765218	0.000000
N	-2.66615722	-2.23335851	0.000000
N	-0.01588271	-2.38671999	0.000000
N	2.61976326	-2.05854722	0.000000
N	4.91807751	-0.69096118	0.000000
N	4.01482335	1.81938964	0.000000
N	1.40244108	2.37112588	0.000000

4. Variettties of HOMO energy, LUMO energy and HOMO-LUMO energy gap of B9N9

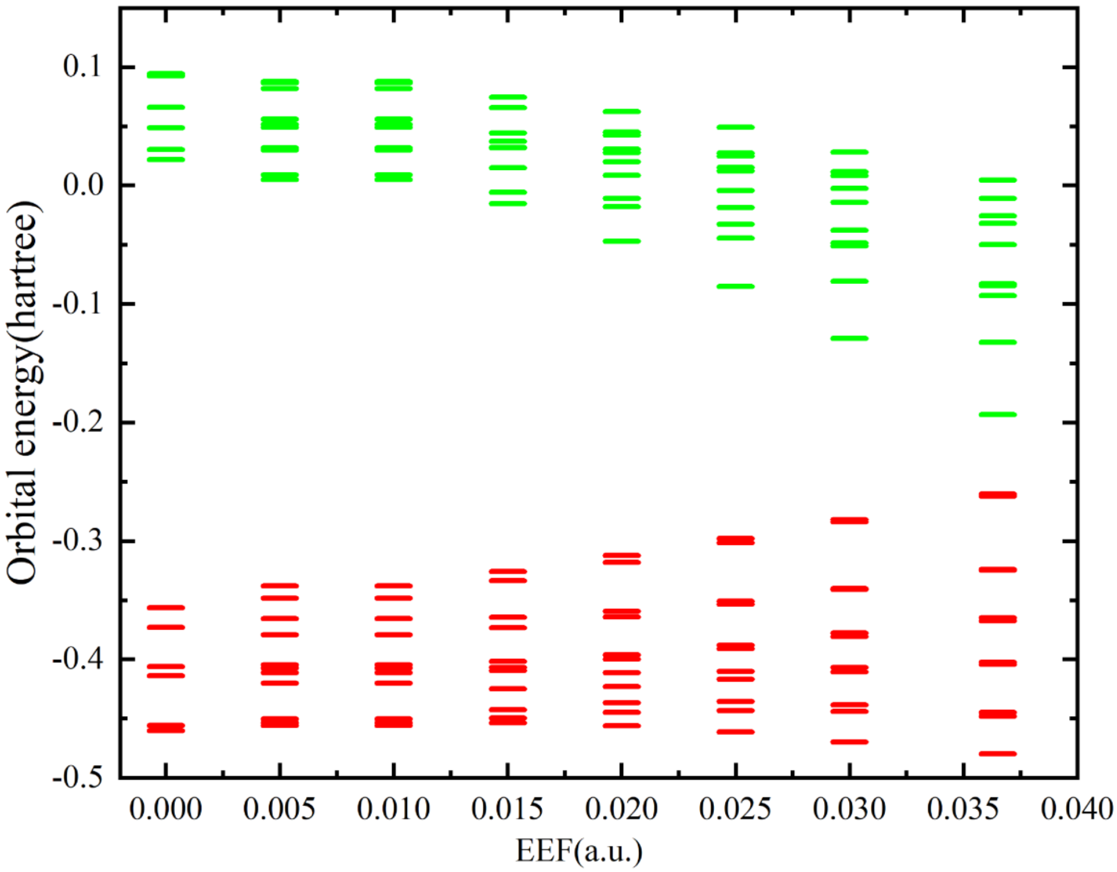

Figure S3 Changes in the HOMO energy and LUMO energy of B_9N_9 under the action of an external electric field. The green generation is LUMO, and the red is HOMO.
